# Comparative Genome Analysis Across 128 *Phytophthora* Isolates Reveal Species-Specific Microsatellite Distribution and Localized Evolution of Compartmentalized Genomes

**DOI:** 10.3389/fmicb.2022.806398

**Published:** 2022-03-16

**Authors:** Kajal Mandal, Subhajeet Dutta, Aditya Upadhyay, Arijit Panda, Sucheta Tripathy

**Affiliations:** ^1^Computational Genomics Laboratory, Department of Structural Biology and Bioinformatics, CSIR-Indian Institute of Chemical Biology, Kolkata, India; ^2^Academy of Scientific and Innovative Research (AcSIR), Ghaziabad, India; ^3^Department of Quantitative Health Science, Mayo Clinic, Rochester, MN, United States

**Keywords:** *Phytophthora*, genome annotation, effectors, RxLRs, simple sequence repeats, motif preference, whole genome duplication, two-speed genome

## Abstract

*Phytophthora* sp. are invasive groups of pathogens belonging to class Oomycetes. In order to contain and control them, a deep knowledge of their biology and infection strategy is imperative. With the availability of large-scale sequencing data, it has been possible to look directly into their genetic material and understand the strategies adopted by them for becoming successful pathogens. Here, we have studied the genomes of 128 *Phytophthora* species available publicly with reasonable quality. Our analysis reveals that the simple sequence repeats (SSRs) of all *Phytophthora* sp. follow distinct isolate specific patterns. We further show that TG/CA dinucleotide repeats are far more abundant in *Phytophthora* sp. than other classes of repeats. In case of tri- and tetranucleotide SSRs also, TG/CA-containing motifs always dominate over others. The GC content of the SSRs are stable without much variation across the isolates of *Phytophthora*. Telomeric repeats of *Phytophthora* follow a pattern of (TTTAGGG)_n_ or (TTAGGGT)_n_ rather than the canonical (TTAGGG)n. RxLR (arginine-any amino acid-leucine-arginine) motifs containing effectors diverge rapidly in *Phytophthora* and do not show any core common group. The RxLR effectors of some *Phytophthora* isolates have a tendency to form clusters with RxLRs from other species than within the same species. An analysis of the flanking intergenic distance clearly indicates a two-speed genome organization for all the *Phytophthora* isolates. Apart from effectors and the transposons, a large number of other virulence genes such as carbohydrate-active enzymes (CAZymes), transcriptional regulators, signal transduction genes, ATP-binding cassette transporters (ABC), and ubiquitins are also present in the repeat-rich compartments. This indicates a rapid co-evolution of this powerful arsenal for successful pathogenicity. Whole genome duplication studies indicate that the pattern followed is more specific to a geographic location. To conclude, the large-scale genomic studies of *Phytophthora* have thrown light on their adaptive evolution, which is largely guided by the localized host-mediated selection pressure.

## Introduction

Oomycetes are one of the most devastating groups of plant pathogens, resembling mostly filamentous fungi. In the post-genomics era, they are placed under stramenopiles that largely include brown algae and diatoms (Kamoun et al., 2015; Derelle et al., 2016; Hannat et al., 2021). A deep knowledge of oomycetes is very important because of its diversity on host preference involving agricultural crops that causes huge economic loss (Marano et al., 2016). *Phytophthora*, the notorious causal agent for the infamous Irish potato famine, is the most common and pathogenic genus of oomycetes that have more than 180 formal species and are abundant in almost all ecosystems (Yang et al., 2017). They are usually soil-borne in nature and have a wide host range causing the root rot, stem rot, blight, and fruit rot of herbaceous and woody plants (Dodds and Rathjen, 2010).

Bioinformatics tools help in assigning functions to the raw genome sequences (Abril and Castellano Hereza, 2019). For large datasets, assigning important features and meaningful biological information to the sequenced genomes helps to characterize them quickly (Stein, 2001). Due to the complexity of the eukaryotic genomes, gene finding is quite a complicated task compared to prokaryotic genomes (Salzberg, 2019). Moreover, for a non-model organism, it is much more difficult due to lack of trained gene models (da Fonseca et al., 2016). Many annotation pipelines are available recently, and the notable ones such as BRAKER2 (Brůna et al., 2021), funannotate^1^, and MAKER (Cantarel et al., 2008) require proper training datasets for predicting gene models. These platforms are mature in predicting gene models and identifying features, but the outcome is extremely unreliable if a trained species dataset is not available. Funannotate, for instance, produces a very poor result if the modeled organism is different from the organism used for prediction. BRAKER2, on the other hand, uses a protein dataset for training and predicts genes using GeneMark-EP + and AUGUSTUS. Easy dissemination of annotation results through data warehouses is also an important area. Over the years, several oomycetes genome resources such as eumicrobedb.org (Panda et al., 2018) and FungiDB (Basenko et al., 2018) have been created and maintained by community members. While both eumicrobedb and FungiDB are primarily based on the genome unified schema (Clark et al., 2005), the portability and ease of handling data are very difficult. Therefore, there is a need for creating resources that can be easily updated.

In *Phytophthora*, genome evolution is influenced by transposable elements (TEs) that give rise to genome fluidity. The genes responsible for pathogenicity, especially the effectors, tend to be localized in TE-rich regions that are gene-sparse regions contrary to the core gene regions, i.e., gene-dense regions (Raffaele and Kamoun, 2012; Engelbrecht et al., 2021). This partitioning of genomes with different evolutionary rates refers to the “two-speed genome” concept.

The mechanisms behind the acquisition and evolution of virulence of *Phytophthora* species is the most studied area for oomycetes biologists. Virulence is controlled by genome architecture, and it subsequently influences the number of specialized effectors that enter the host cells to establish infection (Tyler et al., 2006; Franceschetti et al., 2017). Effectors are broadly categorized into extracellular and intracellular types (McGowan and Fitzpatrick, 2020). Extracellular effectors are secreted on to the host cell and interacted with apoplastic proteins of the host. This includes cell wall-degrading enzymes, protease inhibitors, and elicitins (McGowan and Fitzpatrick, 2017). Intracellular effectors are translated into the host cell and interact with defense-related proteins in the host to manipulate the host immunity. Intracellular effectors are mainly divided into two families, RxLRs (arginine-any amino acid-leucine-arginine) and crinklers (CRN) (Haas et al., 2009). RxLRs are the most abundant family of effectors and are characterized by the presence of conserved amino acid motif arginine (R)–any amino acid (X)– leucine (L)–arginine (R), usually followed by dEER (aspartate glutamate glutamate arginine) domain at their N-terminus (Jiang et al., 2008; Birch et al., 2009). These RxLR-dEER conserved motifs actively participate in translocation, the secretion of effectors into the host cell during the biotrophic phase of infection (Whisson et al., 2007; Schornack et al., 2010; Wawra et al., 2017). The CRN effectors are named after their crinkling and necrosis-influencing activities inside the hosts, which contain conserved LFLAK (leucine phenylalanine leucine alanine lysine) domain at their N-terminal that is associated with translocation into the host cell (Schornack et al., 2010). Effector prediction is a challenging task since they do not generally share features with other protein-coding genes. This is more so since these genes undergo rapid evolution, and therefore, the sequences do not generally possess significant similarity. Another challenge in the prediction of these effectors is due to their location in the genome. Since these effectors are localized mostly at the repeat-rich regions, this region is either unsequenceable or contributes to genome assembly fragmentation. The prediction of effectors from *Phytophthora* is a significant challenge that may lead to the development of disease control strategies.

Simple sequence repeats (SSRs) or microsatellites are the repetition of specific nucleotides distributed in different parts of the genomes and are considered as one the most powerful molecular tools for the identification of inter- and intraspecific variability of genomes (Ellegren, 2004; Gonthier et al., 2015; Biasi et al., 2016). The presence of high DNA replication error and mutation rate in the SSR region in comparison with other parts of the genome produce a high degree of length polymorphism within close organisms (Selkoe and Toonen, 2006; Mascheretti et al., 2008; Olango et al., 2015). The distribution of SSRs in the genome is not random and shows preference toward non-coding regions rather than coding regions due to the selection pressure against frameshift mutations. Although exception is applicable for tri- and hexanucleotide motifs because they do not make frameshift, which supports the fact that the distribution of trinucleotide motif SSRs are higher in coding regions (Tóth et al., 2000; Srivastava et al., 2019). There are many important biological features attributed to SSRs such as codominance, multiallelic nature, and genetic markers just to name a few. These features are used for determining the mating type, genome reconstruction, disease dynamics, and determination of population structures (Schena et al., 2008; Biasi et al., 2016; Stewart et al., 2016; Engelbrecht et al., 2017; Parada-Rojas and Quesada-Ocampo, 2018; Hieno et al., 2019; Zhang et al., 2019; Guo et al., 2021). The available literature indicates studies involving fewer species and lacks a broader view.

In the present study, we have selected 128 assembled *Phytophthora* genomes of reasonable quality from Genbank and annotated 70 genomes whose annotation was not available in Genbank using BRAKER2 (Brůna et al., 2021). We have further compared all the species among themselves using several approaches including genome Mash distance, effector clustering, SSR properties, and whole genome duplication. We have compared the outcomes of SSR clustering and effector clustering with the phylogeny that was already described in Yang et al. (2017) and concurred with the finding. To investigate the evolutionary concept of effector localization in *Phytophthora*, we have performed “two-speed genome” analysis. We have gone beyond to check the other genes apart from effectors, which are localized in gene-sparse regions. We have carried out the two-speed genome analysis using the intergenic distances as a function.

We have further created browsable genome resources and repositories of annotated files to bridge the knowledge gap. We have also shared the annotation and training resources for the ease of annotation of the related species. The genome database is available at www.eumicrobedb.org:3000. All the annotated files such as GFF3, coding sequence (CDS), and protein are deposited into https://zenodo.org/record/5785473#.YcB4D2hBzIV and are now made publicly available. All the scripts and programs used in this study are deposited in https://github.com/computational-genomics-lab/scripts-for-SSR-project.

## Materials and Methods

### Data Collection

The FASTA files of all the publicly available *Phytophthora* genomes of reasonable quality were collected from the Genbank file transfer protocol (FTP) site^2^. A total of 162 genomes were available in the National Center for Biotechnology Information (NCBI) as of July 11, 2021. For analysis, we only took genomes having scaffold level assemblies counting to 128 genomes belonging to 33 species (Supplementary File 1). The assemblies of several genomes were extremely fragmented, resulting in a difficulty in analysis. Therefore, two genomes, *Phytophthora cambivora* isolate: CBS114087 and *P.* x *alni* were not used for further annotation. This made the total number of annotated species to 31 (yellow marked rows in Supplementary File 1 are unannotated). We have created standard genome prefixes by using the first three letters of the genus name (“Phy” in this case) and first two letters of species name (e.g., “so” for sojae), followed by the isolate name separated from the genome prefix with an underscore (“_” For example, Phyin_T30-4 stands for *P. infestans* isolate T30-4).

### Genome Completeness Prediction

Genome assembly completeness was performed for each of the genomes with benchmarking universal single-copy orthologs analysis (BUSCO v. 5.2.2) (Seppey et al., 2019) using BUSCO data set “stramenopiles_odb10.2019-11-21” containing 100 conserved genes. We have used the genomes as well as predicted proteins as the input for BUSCO analysis. The following command was used to run BUSCO:

busco -i <genome input dir> -o <output dir> -m geno -l stramenopiles_odb10 -c 80 2>&1 | tee <logfile name>

### Gene Prediction and Genome Annotation

Out of the 128 available genomes of *Phytophthora*, 56 already had annotations available from Genbank resources. We have therefore carried out gene model prediction using the BRAKER2 pipeline (Brůna et al., 2021) for the 72 remaining isolates. Out of the 72 remaining isolates, *P. cambivora* isolate: CBS114087 and *P.* x *alni* could not be annotated due to fragmented genome assembly.

The draft genome assemblies were first cleaned, followed by doing a soft-masking using redmask v 0.0.2^3^. Then, the soft-masked assemblies were used for gene prediction using BRAKER2. For training, we have used protein files for generating training models and, subsequently, genes were predicted. For the species isolates from NCBI, which have well-annotated protein data, we have used one of the isolate protein data as a protein hint file for the gene prediction of other non-annotated isolates of the same species. For example, for the isolates of *P. capsici*, Phyca_CPV-219.fna, Phyca_CPV-262.fna, etc., we have used the annotated proteins of Phyca_LT1534-B.fna as a hint file. On the other hand, for those species that do not have any annotated isolates available, we have merged all the proteins of previously mentioned 56 annotated genomes and used the merged proteome ‘‘PROTHINT.faa^4^“as a hint file.

Downstream Functional annotation was carried out using funannotate^5^ that includes several databases such as pfam v34.0, uniprot v2021_03, buscoalveolata_stramenophiles, emapper-2.1.4-2-6-g05f27b0, signalp v5.0b, merops v12.0, and CAZy. The completeness of predicted protein sets was further evaluated on BUSCO v5.2.2 using the stramenopiles_odb10 dataset in “prot” mode.

### Phylogenetic Analysis of the Genomes

A phylogenetic tree spanning 128 *Phytophthora* isolates was constructed using Mash distances with the help of Mashtree v.1.2.0 software (Katz et al., 2019). The Mashtree tool used Mash (Ondov et al., 2016) to create MinHash sketches (number of hashed kmers) of the genomes with the help of Mash sketch function with default parameters. Mash distances were then calculated between those sequences using their MinHash sketches, which estimate the mutation rate between them. The more similar genome sequences were likely to share more common MinHashes and less Mash distances. Furthermore, Mash distances were stored in a pairwise distance matrix that was used for building the dendrogram. The neighbor-joining (NJ) algorithm was implemented here. Bootstrapping was performed 1,000 times. The tree was visualized and annotated with the help of iTOL v6 (Letunic and Bork, 2021). The following command was used to run Mashtree.

mashtree_bootstrap.pl –reps 1000 –numcpus 50 *.fna – –min-depth 0 > mashtree.bootstrap.dnd

We have now presented the tree in a circular format with certain annotated features. The features include host preference (curated from available literature), predicted genome features such as genome size (in MB X10 i.e., 100 KB range), the number of predicted effectors, and number of SSRs motifs.

### Whole Genome Duplication Analysis

Ks (or dS) refers to the expected number of synonymous substitutions per synonymous site, also known as synonymous distances between two DNA CDSs. For the detection of whole genome duplication (WGD) events, the whole paranome Ks distributions, constituting all estimated Ks values for all gene duplication events of the genome, were constructed for each of 126 *Phytophthora* genomes using the CDSs with the help of a whole genome duplication-detecting tool wgd v1.1 (Zwaenepoel and Van de Peer, 2018). We have used the kernel density estimation (KDE) model to the Ks distributions and looked for the peaks in the distributions as an evidence of WGD event.

### Genome Binning and Calculation of Flanking Intergenic Regions

We have used in-house perl scripts for determining the intergenic distances flanking the genes in their 5′ and 3′ regions from the BRAKER2-generated GFF3 files or Genbank-annotated files merged with the predicted effector GFF3 files. We have computed the mean, max, and min distances using in-house perl scripts. Most of the effectors are predicted from small fragmented scaffolds; therefore, they had an intergenic distance of zero. We have eliminated such cases from analysis. We have also removed cases where the BRAKER2-predicted gene model overlapped with our predicted effector, resulting in a negative distance between the genes. For computing the significant differences between the 5′ and 3′ Flanking Intergenic Regions (FIRs) of all genes, BUSCO genes, and RxLRs, we have used a two-tailed *t*-test for paired samples. We have plotted the data using Python and R scripts. The scripts are available at: https://github.com/computational-genomics-lab/scripts-for-SSR-project.

### Genome-Wide Simple Sequence Repeat Identifications

For SSR identification, we have taken the exact repetition of motif without any mismatch (perfect SSR), which was identified from the whole genome sequence of all downloaded data using the software package GMATA (genome-wide microsatellite analyzing toward application)^6^ (Wang and Wang, 2016). We have used 2–10 bp motifs for consideration that are repeated at least five times from both the strands. The following command was used to run GMATA:

perl gmat.pl -i *.fna -r 5 -m 2 -x 10

Here, -r 5 implies at least five times repetition; -m 2 and -x 10 indicate the minimum and maximum range of SSR motifs. This has generated four different types of output files with different extensions; *.fms file containing formatted sequences used for SSR identification; *.fms.sat1 file containing a statistical summary of the input sequence(s); *.ssr file with tab-delimited text containing the name and length of the scaffold, start–end position of SSRs, number of repetitions and the corresponding SSR motifs; .sat2 file containing the overall statistics of predicted SSRs. Here in this study, we have referred to the .ssr and .sat2 files as SSR and SAT2 files, respectively.

### Calculation of Basic Simple Sequence Repeat Features

The basic statistics of SSR analysis derived from the GMATA-generated SSR and SAT2 files were used for comparative studies like SSR frequency, GC content, density, and SSR coverage by using in-house Python scripts available at https://github.com/computational-genomics-lab/scripts-for-SSR-project. Here, SSR density implies the number of bases covered by SSRs per Mb of the genome. SSR coverage denotes the percentage of genome covered by SSRs.

### Calculation of In-Frame Frequency of Trinucleotide Simple Sequence Repeat Motifs

In order to identify the SSRs predicted from the whole genome sequences intersecting with the coding regions, we had intersected the SSR files with their corresponding GFF3 files using bedtools v2.26.0 (Quinlan and Hall, 2010). From the bed-intersected files, we have collected the gene IDs that overlapped with the SSR regions. Then, we have calculated the in-frame frequency of the SSR trinucleotide motifs present within the CDS of the same gene. Thereafter, we have calculated the cumulative frequency for each trinucleotide SSR motif across the particular genome using an in-house Python script “SSR_CDS_overlap.py” available at https://github.com/computational-genomics-lab/scripts-for-SSR-project. Finally, we have generated a heatmap based on these values. We have also computed the abundance of trinucleotide SSR motifs exclusively within the coding regions using the GMATA software with the predicted CDSs of 126 annotated genomes as input files. A heatmap was generated using these values subsequently to show the abundance of specific trinucleotides within the coding regions.

### Effector Prediction

A basic pipeline (Supplementary Figure 1) was created for the effector prediction of *Phytophthora*. At first, all the possible open reading frames (ORFs) within the length 150–1,500 nucleotides were extracted from all the six frames of the assembly files using the getorf tool of EMBOSS package v. 6.6.0.0 (Rice et al., 2000). The extracted ORFs were translated in one frame using the transeq tool of the EMBOSS package. These translated sequences were first classified as secretory proteins based on their SignalP v. 5.0b (Armenteros et al., 2019) scores in the N terminus. The secretory proteins were screened for the presence of any transmembrane helices (TMHs) using TMHMM Server v. 2.0 (Krogh et al., 2001). The SignalP containing proteins lacking any TMH was passed through TargetP-2.0 analysis (Armenteros et al., 2019). TargetP predicts the presence of N-terminal pre-sequence based on where they are targeted including signal peptide (SP) (responsible for secretion), mitochondrial transit peptide (mTP), chloroplast transit peptide (cTP), or thylakoid luminal transit peptide (luTP). The ones containing mTP, cTP, and luTP are removed at this step.

EffectorO (Nur et al., 2021) was used to identify the putative effector proteins from the secretome with default parameters. RxLR hmm model ‘‘pf16810.hmm’’ was downloaded from the pfam database (06/08/2021)^7^. An hmmsearch was performed against the pf16810.hmm database for the effectors predicted by the EffectorO program to ascertain the presence of RxLR motifs. For CRN effector prediction, *Phytophthora*-specific CNR proteins were retrieved from NCBI database, followed by multiple sequence alignment using MUSCLE v3.8.31 (Edgar, 2004). A CRN-specific hmm model was built using the hmmbuild tool from HMMER 3.1b2 package^8^. To detect the effectors containing the WYL (tryptophan tyrosine leucine) domain, “WYL_3.hmm” (downloaded on 06/08/2021) from https://pfam.xfam.org/family/PF18488 database was used.

### Orthology Analysis of RxLRs

The outputs from the effector prediction analysis pipeline (Supplementary Figure 1) resulted in a total number of 19,269 RxLR effectors across 128 isolates. Proteinortho v5 (Lechner et al., 2011) tool was used with default parameters for identifying the clusters (95% minimum reciprocal similarity for additional hits; the E-value of 1e-05 for the blastp; minimum percent identity of 25 for best blast hits; minimum coverage of 50% for the best blast alignments). An unweighted pair group method with arithmetic mean (UPGMA)-based species tree was generated for RxLR orthologous clusters of 128 isolates with the help of po2tree.pl program, which is provided with the Proteinortho v5 tool. The tree was further visualized using iTOL v6^9^ (Letunic and Bork, 2021).

### Browsable Annotated Component Development

At Indian Institute of Chemical Biology (IICB), we have created a React-based single-page application. The React framework was chosen as it is easy to create independent reusable components. New functionalities and plugins are easier to incorporate with this framework. The app, which is in its testing version, includes a (Buels et al., 2016) plugin. The app is available at this address: www.eumicrobedb.org:3000.

### Statistical Analysis for Correlation

To establish the correlation between various numerical variables, scatterplots were made using R programming language (Ihaka and Gentleman, 1996). The packages used were “ggplot2” (Wickham, 2011) to plot the scatterplot and “ggpubr” (Kassambara and Kassambara, 2020) to add the Pearson correlation coefficient as well as the *p*-value of the scatterplot. The script for doing so is present in the following Github link: https://github.com/computational-genomics-lab/scripts-for-SSR-project under the folder R-scripts.

## Results and Discussion

### Number of Predicted Genes Are Co-related With the Genome Size

Out of the 128 genomes studied, 126 genomes had more than 90% BUSCO completeness. However, two species, e.g., *P. cambivora* isolate: CBS 114087 and *P.* x *alni* had extremely fragmented assemblies with 72,332 and 1,184,74 scaffolds, respectively. This exceeded the number of sequences that can be handled in BRAKER2; therefore, we could not annotate these two strains. The genome size and the number of predicted genes in the *Phytophthora* species are correlated (*r* = 0.409, *p*-value 0.00001). The highest number of proteins were predicted (36,721) from *P. syringae* BL57G having a genome size of 74.93 Mb, whereas the lowest was predicted from *P. kernoviae Chile4*, Phyke_Chile4 with a genome size of ∼37 Mb. Other isolates of *P. kernoviae* remain the genomes with the least number of genes (<10,500) that also had the least genome size (<37 Mb).

Since the genomes studied were near 90% complete, it is fair to assume that the number of predicted genes represent 90% of the genes. *P. kernoviae* have the least genome sizes (36--38 Mb) followed by *P. ramorum* species. Both *P. kernoviae* and *P. ramorum* infect tree species and are mostly homothallic and biotrophic^10^,^11^. The species with larger genomes are *P. infestans* and *P. cambivora*. Both the species are heterothallic and have undergone transposon mediated genome expansion (Haas et al., 2009). While it is difficult to establish the link between the genome size and the virulence, it is a well-established fact that organisms having larger genomes and larger gene repertoire, is due to their heterothallic nature. While biotrophs such as *Hyaloperonospora arabidopsidis* are known to have streamlined genomes (Baxter et al., 2010), hemibiotrophs have larger genomes.

### Genome Size and Number of Simple Sequence Repeats Are Positively Correlated

**FIGURE 1:**
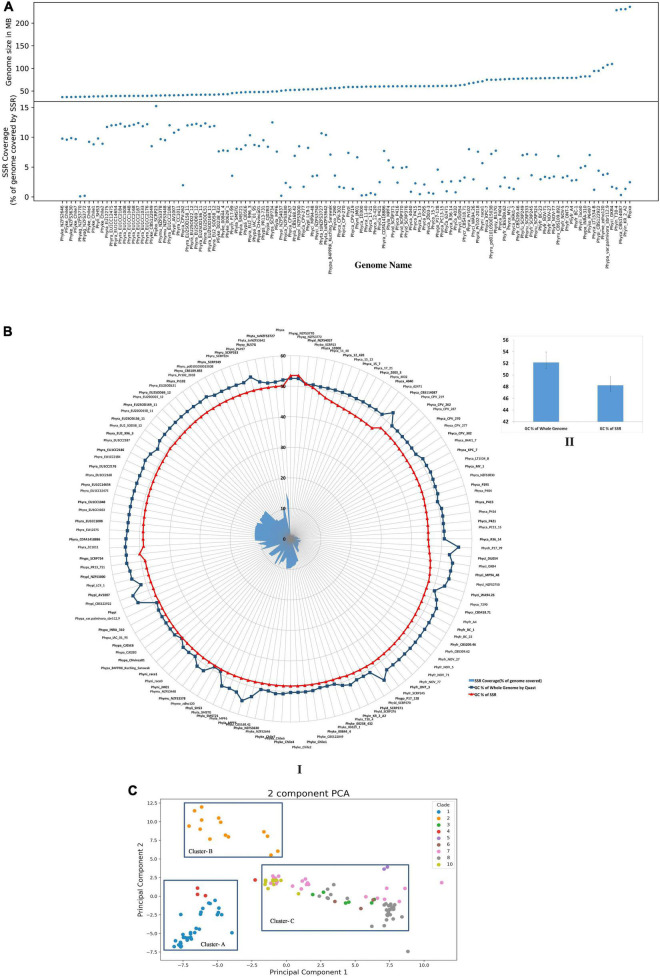
Comparison of various SSR attributes within genomes. **(A)** Comparison of genome size and SSR coverage. **(B)** GC content of SSRs and their corresponding genomes. Genomic GC content of *Phytophthora* (52.13 ± 1.18%) and GC content of SSRs is (48.22 ± 1.5%). I: Is the circular representation of GC content. II: Represents the bar plots of mean genome GC content and SSR GC content. **(C)** PCA clustering using SSR motifs. Each dot represents a genome and is colored based on their clades as described in Yang et al. (2017). Di, tri, and tetramer motifs were taken from each genome, and PCA was done. Genomes from each clade clusters together more than others indicate phylogeny-based SSR variation. Cluster-A contains genomes from clade 1 and 4. Clade 2 positioned separately as Cluster-B. The rest of the genomes from clades 3, 5, 6, 7, 8, and 10 are clustered together as Cluster-C.

We have carried out SSR finding with 2–10 bp units repeated at least five times in all the 128 genomes. This resulted in 391,318 SSRs. The number of SSRs in the genomes is positively correlated with the genome size (Supplementary Figure 2A, *R* = 0.84, *p* = 2.2 e-16). All the SSR files containing microsatellite data and SAT2 files containing information regarding SSR statistics are publicly available at https://zenodo.org/record/5785473#.YcB4D2hBzIV. Studies with mosquitos and other species also reveal that SSR frequencies are directly co-related with the genome size (Srivastava et al., 2019). In order to rationalize the number of bases in SSRs per Mb of genome, we have computed the SSR density. *P. agathidicida* isolate-NZFS3770 had the lowest SSR density (921.81 bp/Mb of genome) and *P. boehmeriae* isolate SCRP23 had the highest density (152,348.35 bp/Mb of genome). The genome sizes of *P. agathidicida* NZFS3770 and *P. boehmeriae* SCRP23 are 37.23 and 39.96 Mb, respectively, which is low compared to other *Phytophthora* genome sizes (Supplementary File 1). Contrary to the number of SSRs and genome size correlation, the genome size and density of SSRs have very little correlation (Supplementary Figure 2B, *R* = −0.19, *p* = 0.034). The percentage of SSRs per genome or genome coverage is another way of depicting SSR density. As expected, *P. boehmeriae* isolate SCRP23 has 15.23% of the genome covered with SSRs. This is followed by *P. ramorum EU* isolates (11%–12%). *P. agathidicida* NZFS3770 has lowest coverage with 0.09% (Figure 1A and Supplementary File 2). Among the *P. ramorum* isolates, the ones isolated from EU (European Union) had significantly higher number of SSRs than that of the American isolates, NA1 strain Pr102 and CDFA1418886 (11%–12% vs. 7%). The European strains of *P. ramorum* are more aggressive than the original NA1 strains found infecting coastal districts of California, United States. Since microsatellites mutate 10 orders of magnitude greater than commonly occurring point mutations (Gemayel et al., 2012), it could be speculated whether increased frequencies of SSRs in virulent isolates indicate greater adaptability. Gain and loss of gene functions are attributed due to frameshift mutations and subsequent fixation. The presence of a higher number of SSRs could possibly mean that the genomic region is in a state of flux and may contribute to adaptation, leading to increased virulence. On the contrary, *P. agathidicida* isolate NZFS3770 having the least SSR density is an extremely virulent pathogen in Kauri (*Agathis australis*) (Studholme et al., 2016).

### The Lower-Order Simple Sequence Repeat Motifs Represent the Major Class in All Isolates

The dinucleotide SSRs are the most abundant class with an average of 66.10% followed by trinucleotide (29.42%) and tetranucleotide SSRs (2.35%). Altogether, the di- tri-, and tetra nucleotide SSRs constitute 97.89% of total SSRs, while the remaining are the penta- to decanucleotide repeats. For all the species, the abundance rank of SSRs are always dimer > trimer > tetramer. The higher-order SSRs do not follow any trend and are mostly species and isolate dependent (Supplementary File 3). The average number of SSR motifs across the isolates is 154.81 with the lowest and highest number of motifs in *P. palmivora* isolates B4_PPRK (90) and *P. cactorum* isolate P404 (286), respectively. It was noticed that the number of SSR motifs are negatively correlated with their genome size, although the correlation is not strong (Supplementary Figure 2C, *R* = −0.12, *p* = 0.18). Out of 128 genomes, 54 had no decanucleotide SSRs, 37 had no octameric SSRs, and 3 without heptameric SSRs.

SSR motif lengths were classified into two categories, Class I (SSR length ≥ 20 base pair) and Class II (SSR length < 20 base pair) and presence of Class I SSRs indicate hyperpolymorphism. We found that all genomes currently studied show more than 80% class II category SSR. Class II SSRs tend to be less variable compared with Class I due to low probability of slipped-strand mispairing on the short SSR strand (Temnykh et al., 2001). Thus, Class I SSRs are better for polymorphism identification than Class II. So, designing markers from Class I SSRs for the identification of polymorphism among the genotypes of a species could be more reliable.

In order to establish the relationship between genomic GC content and SSR GC content, we have computed the GC content for genomic DNA and SSR regions of the genome. While the genomic region had 52.13 ± 1.18% GC, SSR region had comparatively lower 48.22 ± 1.5% GC content (Figure 1B). Statistical analysis shows that there was positive correlation between genomic and SSR GC content (Supplementary Figure 2D, *R* = 0.39, *p* = 7.1 e-06). The GC contents of the *Phytophthora* SSRs are in fact a characteristic feature for the species. Chlorophytes are characterized by GC-rich SSRs; most fungal species are reported to have intermediate GC- containing SSRs, while complex genomes such as plants carry high AT content in SSR (Srivastava et al., 2019).

In order to establish the relatedness of species and clade (Yang et al., 2017) on the basis of SSRs, we have performed principal component analysis (PCA) for di-, tri-, and tetranucleotide motifs from all the genomes. Results indicate a strong species-specific distribution of SSR motifs. PCA shows three clusters (Figure 1C). Clade 1 and clade 4 are present within cluster-A, which indicates that they are close to each other. This fact was already established from phylogenetic analysis, which was based on seven nuclear genetic markers as described by Yang et al. (2017). Only clade 2 represents cluster-B. Cluster-C contains the highest number of isolates and represents clades 3, 5, 6, 7, 8, and 10. From the clustering, it was demonstrated that clade 2 has more distinct SSR motifs than others. Our analysis indicates that the SSR composition of clades 3, 5, 6, 7, 8, and 10 (Cluster C) is closer with each other than with other clades (Cluster A and Cluster B).

### “TG/CA” Dinucleotide Motifs Represent the Most Abundant Class of Simple Sequence Repeats Across All *Phytophthora* Genome Isolates

**FIGURE 2:**
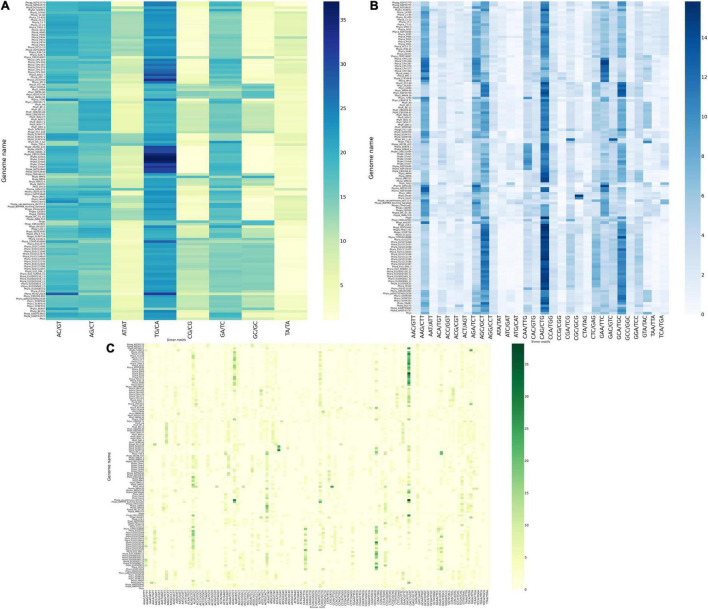
Construction of heatmap of SSR motifs. **(A)** Heatmap of dinucleotide motifs. Out of eight group motifs, TG/CA motif was the most preferred motif in all the genomes and *P. kernoviae* contains the highest percentage of motifs among all the *Phytophthora* genus. Scarcity of AT- containing motif is clearly visible. **(B)** Heatmap of trinucleotide motifs. CAG/CTG motif preference is found in maximum number of genomes. Only a few trinucleotide motifs are predominant, while clearly, the AT-containing motifs such as ATG/CAT and ATC/GAT are very scarce. **(C)** Heatmap of tetranucleotide motifs. There is a clear dominance of GACA/TGTC- containing motif, which has a TG/CA pattern embedded in it.

The occurrence of dinucleotide, trinucleotide, and tetranucleotide SSRs were plotted using heatmap, which constitutes 97.89% of entire predicted SSRs’ cumulative length (Figure 2A). For this, we took two complementary motifs as groups since strandedness is unknown. We have calculated the percentage of the motifs for each class e.g., di-, tri- and tetra-. For example, *P. agathidicida* isolate NZFS3770 contains 1,170 dimeric SSRs and the TG/CA motif is present 294 times, therefore making it 25.12% of the total number of dimers. The dinucleotide SSR motifs are often used as molecular markers due to their higher mutation rates than other types of SSRs (Karaoglu et al., 2005). TG/CA is the most preferred di nucleotide motifs (more than 23% in average among all dinucleotides) as well as among all SSR motifs (more than 15% in average). This pattern was observed in all the 33 species without a single exception; so, it can be concluded that TG/CA motifs are the characteristic features of genus *Phytophthora*. In several studies, species-specific preference for a particular motif for a genus like *Fusarium, Aspergillus*, and *Nicotiana* has been discussed (Mahfooz et al., 2015, 2016; Wang et al., 2018). The second highest percentage of motifs are AC/GT and AG/CT, which occupied 17.86 and 17.24% respectively. This is followed by GA/TC (15.79%), AT/AT (7.55%), GC/GC (7.54%), CG/CG (6.63%), and TA/TA (4.38%) motifs. Presence of TG/CA and AG/CT in higher percentage may be due to their amenability to low mutation rate (Guo et al., 2009). It has been reported that TG/CA and AC/GT were predominant motifs in the mammalian system and AT/AT and TA/TA were abundant in the plant systems (Lagercrantz et al., 1993; Morgante et al., 2002). Dinucleotide motif composition of *Phytophthora* follows opposite to the plant system and contains low amounts of AT or TA motifs. This attribute has been the basis behind separating the contaminating plant DNA from oomycetes DNA (Tripathy et al., 2012).

### Tri and Tetranucleotide Motifs Containing “TG/CA” Patterns Represent the Most Frequent Class of Simple Sequence Repeats

Out of 30 possible groups of “Tri” motifs, 6 motifs (AAG/CTT, AGA/TCT, AGC/GCT, CAG/CTG, GAA/TTC, and GCA/TGC) are present predominantly and occupy 47.1% of total trinucleotide-containing SSRs (Figure 2B). CAG/CTG motifs are found to be the dominant class in 20 *Phytophthora* species that represent 11.27% of total trinucleotide SSR motifs. *P. colocasiae, P. idaei, P. multivora, P. palmivora, P. plurivora*, and *P. parasitica* on the other hand have AAG/CTT as the most predominant motif, similar to fungi *Trichoderma atroviride, T. virens, Aspergillus nidulans* and *A. oryzae* (Mahfooz et al., 2016, 2017). *P. capsici, P. megakarya*, and *P. litchii* show GAA/TTC dominance. At the same time, ATG/CAT and ATC/GAT are the lowest common motifs with less than 1% occurrence.

It has been found that occurrence of trinucleotide SSRs on ORF and 5′- UTR regions was much higher than the non-coding regions of the genome (Gonthier et al., 2015). Thus, motif dominance, which represents a complete codon for trinucleotide SSRs, makes sense that it has an important role in molecular mechanism. Amino acids encoded by the most abundant motifs (CAG/CTG) are leucine and glutamine, AAG/CTT encodes leucine and lysine, respectively. In order to find out the presence of trinucleotide motifs in coding region, we have used CDS file as an input for SSR identification and the result exhibited same pattern with dominance of above mentions groups motifs (Supplementary Figure 3). The presence of trinucleotide on the coding region is not enough for translation, so, we performed overlap of predicted SSRs falling in-frame with the coding regions. The heatmap of in-frame analysis also shows dominance of CAG, CTG, and AAG motifs (Supplementary Figure 4). CAG and AAG codes for leucine amino acid. It has been reported that amino acid leucine helps in zoospore germination, which eventually helps in the establishment of infection to the host of *Phytophthora* (Jiang et al., 2019). Thus, SSRs with leucine CDS may have a vital role in germination, but further in-depth study is required for establishing this link. Basic amino acid lysine and arginine induced encystment in *P. cinnamomi* (Byrt et al., 1982). This may be the reason why these motifs are conserved across all the *Phytophthora* species. Other amino acids encoded by the highly abundant motifs are serine, alanine, arginine, glutamic acid, phenylalanine, and cysteine.

Among the tetranucleotide motifs, GACA/TGTC motif was the most commonly occurring motif among all the 128 genomes studied and occupied 7.45% of the total tetranucleotide SSRs. This was followed by ACAG/CTGT (5.15%), AGTG/CACT (4.16%), and AGAC/GTCT (4.07%) (Figure 2C). These observations further strengthen the predominance of TG/CA dinucleotide that forms a part of the tri- and tetranucleotide motifs, representing the major class.

### Higher-Order Simple Sequence Repeat Motifs Are Specific to Individual *Phytophthora* Species

The higher-order motifs such as the tetra- to decanucleotide repeats are unique for each of the isolates (Table 1). It is also noteworthy that 8 dinucleotide and 22 out of 30 trinucleotide motifs are common in *Phytophthora* genomes and possibly are characteristic features of *Phytophthora* species.

**TABLE 1:** Common SSR motifs found across all the *Phytophthora* isolates.

TG/CA AC/GT GA/TC CT/AG AT/AT CG/CG GC/GC TA/TA CTG/CAG CAA/TTG	GCT/AGC TGC/GCA TCT/AGA TTC/GAA CTT/AAG CGA/TCG GAG/CTC GTC/GAC AAC/GTT TGT/ACA	CAC/GTG TGA/TCA CCA/TGG TCC/GGA ACC/GGT ACG/CGT GTA/TAC AGG/CCT AGT/ACT AAT/ATT

Out of the 128 genomes, 110 genomes have their unique motif containing SSRs, which is specific only to them regardless of the species (Supplementary File 4). *P. colocasiae* isolate-7290 contains the highest number of unique motifs (81), followed by *P. cactorum* isolate-P404 (46), followed by *P. cinnamomi* isolate-GKB4 (32).

For species studied with higher numbers of isolates, the number of species-specific SSR motifs were less and isolate specific SSR motifs were higher (Supplementary File 5). For example, in the case of *P. capsici* (10 isolates), *P. fragariae* (11 isolates), and *P. ramorum* (23 isolates) had no common species specific motifs. In case of *P. cactorum* (18 isolates) has only a single common motif. This possibly indicates that SSR markers are more isolate specific. So, a unique SSR for each isolate can be used to design isolate-specific SSR markers.

### Telomeres Contain an Extra “T” in Addition to the Canonical Telomeric Repeat TTAGGG

For telomere-like sequence identification, we have manually searched for an ancestral telomere motif TTAGGG by Notepad + + software on the GMATA derived SSR data with a minimum of three repeats. Interestingly, it was found that the TTAGGG motif was present with an extra T (thymine) on the start or end position of the motif, e.g., (TTTAGGG)_n_ or (TTAGGGT)_n_. Most of the time these motifs were located at the start or end regions of a scaffold, indicating the end of the chromosome (Supplementary File 6, sheet 1 and sheet 2). A previous study by Fulnečková et al. (2013), reported that (TTTAGGG)_n_ is a characteristic feature of telomere sequence for plants and oomycetes while mammals and fungi have (TTAGGG)_n_.

### Absence of Core RxLR Clusters Is an Indication of Their Rapid Divergence

The standard effector prediction pipeline (Chepsergon et al., 2021) was used to predict the effectors. The number of proteins retained in each step is shown in Supplementary Figure 6. The percentage of RxLR effectors were much higher than other predicted class of effectors as it is primarily associated with infection of host and a recent study also gives same indication (Gao et al., 2021). A significantly higher amount of RxLR effectors containing species are *P. megakarya, P. palmivora, P. cambivora, P. ramorum* (isolate- Pr102 and Pr102-2018), *P. infestans, P. nicotianae, P. sojae*, etc. On the other hand, the lowest number of RxLR was found in *P. pisi* followed by *P. chlamydospora*, *P. syringae* (Supplementary File 7). For CRN motifs containing effectors, *P. infestans* and *P. sojae* have the highest number. A higher number of CRN effectors are an indication of preference toward necrotrophic life cycle (Stam et al., 2013).

**FIGURE 3:**
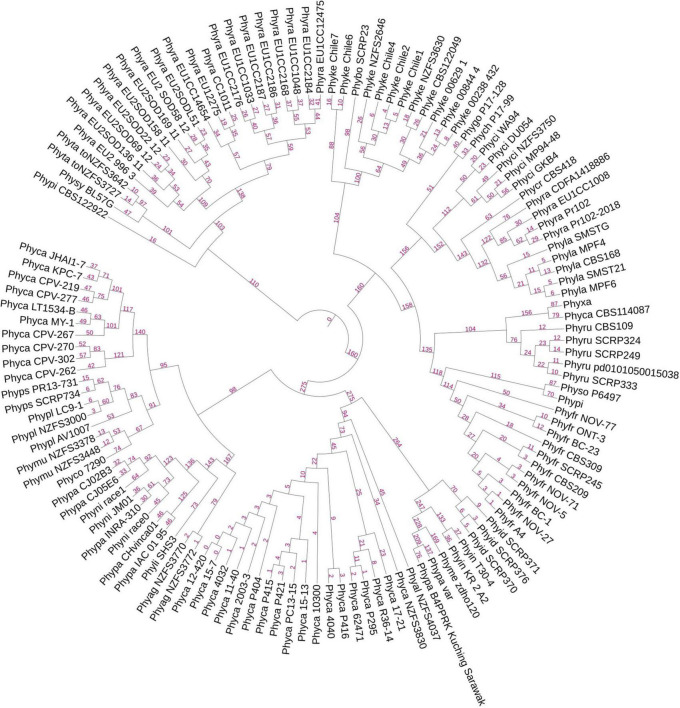
A UPGMA-based rooted species tree showing placement of 128 genomes on the basis of RxLR orthologous clusters. The branching lengths are shown in pink color on the branches of the tree. Species-specific distribution of RxLR effectors was found in most of the cases. There are exceptions where some species have closeness to other species than within themselves. Examples are species *P. parasitica*, *P. nicotianae*, and *P. kernoviae*.

**FIGURE 4:**
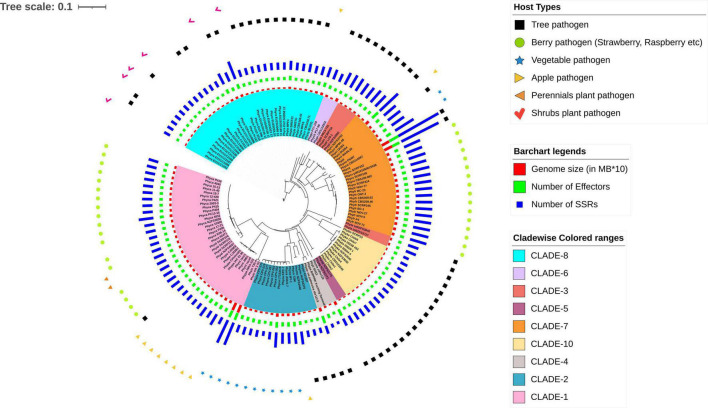
A rooted phylogenetic tree showing evolutionary relationships and the connections between the 128 *Phytophthora* isolates. The tree is generated using NJ methods in the Mashtree software. The genomes are highlighted in different colors according to their clades as described by Yang et al. (2017) and are as follows: cyan, CLADE-8; light violet, CLADE-6; deep pink, CLADE-3; deep violet, CLADE-5; orange, CLADE-7; pale yellow, CLADE-10; gray, CLADE-4; blue, CLADE-2; and light pink, CLADE-1. Different genome features are represented in the form of bar charts such as red bar plot for genome size (in MB*10), green for number of effectors, and violet for number of SSRs. The host types for the *Phytophthora* pathogens are shown using different symbols over the bar charts such as black square for Tree pathogen, green circle for Berry pathogen (strawberry, raspberry, etc.); blue star for Vegetable pathogen; pale-yellow right-sided triangle for Apple pathogen; orange left-sided triangle for Perennials plant pathogen; and pink tick for Shrubs plant pathogen.

For the prediction of core RxLR effectors (CRE), we took all the RxLR effectors and ran ProteinorthoV5 through them in order to detect orthologous genes within different species. This resulted in 1,461 orthologous clusters. The singletons containing single RxLRs were discarded and were not considered for further analysis (Supplementary File 8). The largest cluster contained 150 proteins representing 105 isolates, which indicates the presence of co-orthologs. We could not predict any core ortholog common across all the 128 isolates studied. Further, we have built a UPGMA species tree on the basis of the clustering patterns of the effectors (Figure 3). In most of the cases, the effectors are species specific and isolates of the same species clustered together (Figure 3). There are exceptions in cases of *P. ramorum*, where the RxLRs make two distinct groups. Group 1 contains isolates-CDFA1418886, EU1CC1008, Pr102, and Pr102-2018 that are more close to *P. lateralis*, whereas other 19 isolates of *P. ramorum* (Group-2) had more similarity with *P. taxon totara* and *P. syringae*. Another exception is *P. kernoviae* where isolate- Chile 6 and Chile 7 makes a different group from the other isolates of the same species. *P. parasitica* isolate INRA-310 and *P. nicotianae* isolate JM01 make a closer group than other isolates of their own species. The genome Mash distances (Figure 4) have grouped the isolates from individual *Phytophthora* species together. However, clusters based on RxLRs grouped isolates of *P. ramorum* into two distinct groups. One containing NA1 isolates from the United States and the other one containing the EU isolates. Similarly, in the case of *P. kernoviae*, two distinct groups were formed. The evolution of the pathogenicity of *Phytophthora* is very complex and driven by many factors that are heavily dependent on host preference. It is therefore not clear if the RxLRs bearing close similarity among other species is an evolutionary strategy for survival.

### Flanking Intergenic Region Distance Indicates Clear Two Speed Genome Architecture in All the *Phytophthora* Isolates

**FIGURE 5:**
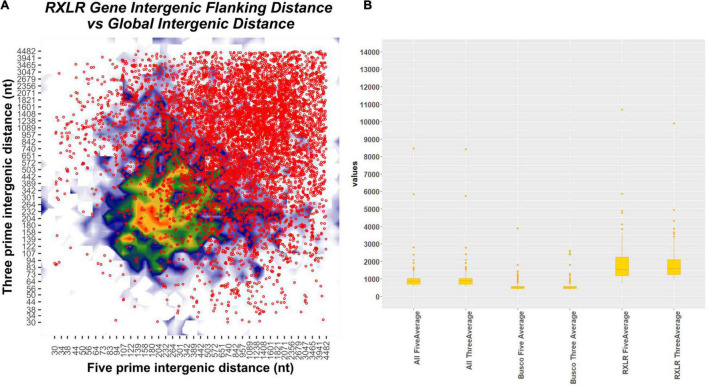
Plot showing flanking intergenic distances of different categories of genes of all the 128 *Phytophthora* species concatenated together. (A) Contour plot depicting the five prime and three prime intergenic distances plotted in x- and y-axis. The scatter plot on the top of the contour plot in red dots depicts the position of the RxLR effectors overlaid on the contour plot. There is a clear indication that the RxLRs have significantly large intergenic distances than the other genes. (B) Box plot of intergenic distances (both 5′ and 3′ regions) of all the genes concatenated together with BUSCO genes and RxLR genes. The position and the median point of the boxes are indicators of higher 5′and 3′ distances seen in all the *Phytophthora* genomes.

We have computed the intergenic distances of the genes in each of the species having predicted gene models (No # 126) (Supplementary File 9). The distances and their mean values are provided in Supplementary File 9. We have conducted a two-tailed *t*-test for paired samples for comparing the average values of flanking 5′ distance of the genes and 3′distance of the genes. The average 5′ FIRs and the 3′ FIRs among all the species did not have any significant difference (*p*-value = 0.919; for BUSCO genes = 0.931; for RxLR effectors = 0.81). However, between the 5′ genomic distance of all genes with RxLRs, the *p*-value is 1.232^7^ and the 3′ distance is 7.93^–8^. The average 5′ intergenic distance between BUSCO genes and the RxLR have a *p*-value of 1.68^–16^ and the 3′ distance is 3.68^–18^ (Supplementary File 9, Supplementary Figure 6, and Figure 5). This confirms the two-speed genome theory involving the RxLR effectors.

We have further curated the annotations of the 1,000 genes that are placed at the extremely sparse genomic locations in each of the *Phytophthora* genomes (total # 126 × 1,000 = 126,000 genes). It is interesting to note that out of the 126,000 genes studied, 123,520 had annotations. 42.61% (52,643) are annotated as hypothetical proteins, without any known functions. Among the annotated proteins, 1,004 are RxLR proteins, 255 are CRN, and 263 are elicitins. Among others, the most notable ones are carbohydrate-active enZYmes (CAZymes) such as peptidases (288), pectin esterases (205), and glycosyl transferases (137) (see text footnote 4). Among the other categories are the transcription factors, CW-ype zinc finger protein, CXXC motif-containing genes, EF–hand proteins, PWWP domain- containing protein, calcineurins, ubiquitins, etc. Signal transduction proteins such as WD40 are in high numbers in the gene-sparse regions. We have located thousands of transposons and retroposons in the gene- sparse regions of the 126 *Phytophthora* species.

Numerous reports are available to suggest that the oomycetes pathogens have rapidly evolving powerful arsenals that are used to combat the host defense mechanisms. Pathogens combat hosts with the choicest effectors that are not randomly distributed in the genome. Rather, they are localized in regions rich in transposons and repeats (Dong et al., 2015). Many oomycete organisms have already been studied with a robust two-speed genome composition, where the effectors are located in gene- sparse regions (Vetukuri et al., 2018; Malar et al., 2019). However, there is a lack of extensive studies on the overall composition of the gene-sparse regions. We have analyzed the 1,000 genes of each genome located in the most gene-sparse regions in all the 126 genomes under study. As expected, the genes involving pathogenesis are enriched in this region. Apart from that, many regulatory genes, transcription factors, and signal transduction genes are located in these areas (see text footnote 4).

### Two Types of Genome Duplication Events Occur in *Phytophthora*

**FIGURE 6:**
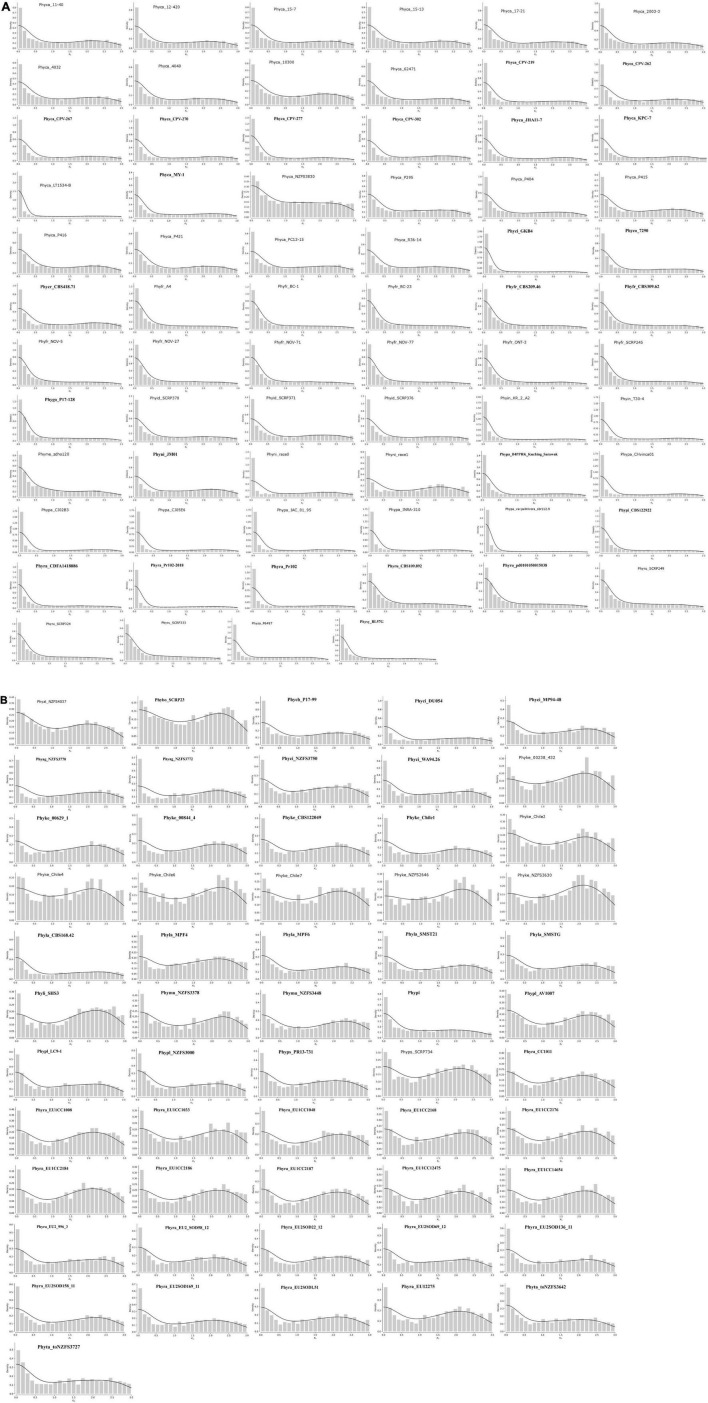
K_s_ distribution of full paranomes. (A) Type-I whole genome duplication with KDEs of peaks in the K_*S.*_ Exponential decrease represents L-shaped curve that indicates whole genome duplication in ancestral time and loss of many duplicate genes. (B) Type-II whole genome duplication with KDE. Peaks in the K_s_ at 2–2.5 indicate increase of duplication frequency.

Genome duplication analysis was conducted using the methods described in Zwaenepoel and Van de Peer (2018). Small-scale duplication and the loss of duplicated copies are not under selection pressure and are a continuous process. However, if large-scale duplication events occur, i.e., whole genome duplication (WGD), then it is visible as peaks in the number of retained duplicates (Zwaenepoel and Van de Peer, 2018). We have used kernel density to show genome duplication events in *Phytophthora* species. Our results show various levels of genome duplication in all the isolates. Interestingly, we have observed two types of genome duplication patterns and we have categorized them as type I and type II. In the case of type I, whole genome duplication happens once at K_s_ 0–0.5, and then the numbers of duplicated genes decrease gradually due to lesser selection pressure, giving an L-shaped distribution to the graph, although a sufficient number of duplicated genes are present (Figure 6A). In type II, the pattern of duplication is quite different from type 1 where we have observed the presence of distinct peaks at higher K_s_ 2.0–2.5. This kind of pattern occurs due to the increase in duplication frequency (Figure 6B).

All isolates of *P. capsici, P. cactorum, P. fragariae, P. idaei, P. infestans, P. megakarya, P. palmivora, P. parasitica, P. nicotianae, P. rubi, P. sojae, P. pinifolia, P. colocasiae, P. cryptogea*, and *P. cryptogea* show type I WGD. Previous studies provide evidence for the presence of ancestral WGD in *P. capsici* (Cui et al., 2019), *P. cactorum* (Yang et al., 2018), *P. infestans*, and *P. sojae* (Martens and Van de Peer, 2010). Recently, Morales-Cruz et al. (2020) investigated the whole genome duplication of two species, e.g., *P. megakarya*, and *P. palmivora*. They have demonstrated that both the species go through independent WGDs, which results in large genome size with a higher number of RxLR, CRN, and other pathogenesis-related genes. Type II WGD was noticed in all isolates of *P. agathidicida, P. kernoviae, P. lateralis, P. litchi, P. multivora, P. pisi, P. plurivora, P. pluvialis, P. pseudosyringae, P. chlamydospora*, and *P. taxon totara.* It was also noticed that different isolates of the same species exhibited different types of WGDs. We studied WGD in all 22 isolates of *P. ramorum* where three isolates show type I WGD and rest show type II WGD. The type I WGD-containing isolates of *P. ramorum* are from the United States (Phyra_CDFA1418886, Phyra_Pr102, and Phyra_Pr102-2018), whereas type II-containing isolates are from Europe. We have also shown that the type I-containing ramorum isolates have a lesser number of SSRs than the type II-containing ramorum isolates from Europe. *P. cinnamomi* isolates GKB4 show type I WGD and have a genome size of 106 MB, whereas other isolates, i.e., DU054, MP94-48, NZFS3750, and WA94.26 have type II WGD and their genome size is nearly half the size of GKB4. *P. aleatoria* and *P. boehmeria* also have type II WGD, but two strong peaks were observed, which might be the due to the duplication happening in different time points.

WGDs can result due to autopolyploidy or by allopolyploidy, and both the events have been reported in *Phytophthora* (Redondo et al., 2015). WGD followed by gene loss plays a major evolutionary force to gene sub-functionalization and neo-functionalization in plants and animal systems (Huang et al., 2013). Our analysis suggests that the levels of genome duplication are largely due to their genome localization in a specific geographical region and the selection pressure acting upon them. *Phytophthora* adapts to host-induced selection pressure by genome rearrangements and expansion mediated by repeats (Kamoun et al., 2015). Thus large-scale duplication events increase pathogen fitness in a given environment and specific host that is clearly evident from this analysis (Redondo et al., 2015).

## Conclusion

The analysis of 128 *Phytophthora* genomes isolated from various geographical locations indicates that there is localized genome evolution and genome duplication. SSR motifs are preserved in an isolate-specific manner and can act as a unique identifier for a certain isolate. All the isolates of *Phytophthora* adhere to genome compartmentalization, where the core genes occur in compact regions of the genome. The infection-related genes and genes responsible for adaptive evolution, on the other hand, are localized in more repeat-rich regions amenable to rapid changes. All the annotated data and associated files are publicly deposited for community consumption. The browsable genomes and their annotations are available in www.eumicrobedb.org:3000.

## Data Availability Statement

The datasets presented in this study can be found in online repositories. The names of the repository/repositories and accession number(s) can be found in the article/Supplementary Material.

## Author Contributions

ST conceived the project. SD, KM, ST, AP, and AU carried out the data analysis. AU done the database design and upload with the help of AP. ST, KM, SD, and AU wrote the manuscript. All authors read and agreed on the contents of the manuscript.

## Conflict of Interest

The authors declare that the research was conducted in the absence of any commercial or financial relationships that could be construed as a potential conflict of interest.

## Publisher’s Note

All claims expressed in this article are solely those of the authors and do not necessarily represent those of their affiliated organizations, or those of the publisher, the editors and the reviewers. Any product that may be evaluated in this article, or claim that may be made by its manufacturer, is not guaranteed or endorsed by the publisher.

## Footnotes

1
https://github.com/nextgenusfs/funannotate

2
https://www.ncbi.nlm.nih.gov/data-hub/taxonomy/4783/?utm_source=assembly&utm_medium=referral&utm_campaign=KnownItemSensor:taxname

3
https://github.com/nextgenusfs/redmask

4
https://zenodo.org/record/5785473#.YcB4D2hBzIV

5
https://github.com/nextgenusfs/funannotate

6
https://sourceforge.net/projects/gmata/

7
http://pfam.xfam.org/family/PF16810

8
http://hmmer.org/

9
https://itol.embl.de/

10
https://www.cabi.org/isc/datasheet/40972

11
https://www.cabi.org/isc/datasheet/40991

## Supplementary Material

Supplementary Figure 1 Overall pipeline used for secretome and effector prediction.

Supplementary Figure 2 (A) Pearson’s correlation coefficient of SSR number and Genome size. Result indicates number of SSRs positively correlated with genome size. (B) There was weak negative correlation between SSR density and genome size. (C) Number of SSR motifs shows weak negative correlation with genome size. (D) GC percentage of genome and SSR are positively correlated.

Supplementary Figure 3 Heatmap depicts the percentage frequency of trinucleotide SSR group motifs in the coding sequences (CDS). There is a clear abundance of CAG/CTG, AAG/CTT motifs.

Supplementary Figure 4 Heatmap depicts the in-frame cumulative frequency of trinucleotide SSR motifs in the coding sequences (CDS). There is a clear dominance of CAG, CTG, AAG motifs.

Supplementary Figure 5 Effector prediction pipeline containing the number of proteins filtered out in each step. The first bar chart (bottom most) (light green) represents the genome sizes of 128 Phytophthora isolates studied. The second bar chart (ash) represents the total number of Open reading frames (ORFs) predicted by getorf function of EMBOSS package. These ORFs are further translated in one frame and the translated sequences were used for secretome prediction. The third bar chart (pink) shows the number of secretory proteins containing signal peptide (SP), based on SignalP v. 5.0b prediction. The fourth one (brown) shows the number of secretory proteins retained after the TMHMM analysis that does not contain any TransMembrane Helices (TMHs). The fifth one (violet) shows the number of secretory proteins that passed TargetP analysis. These retained proteins after the TargetP are used for effector prediction using EffectorO. The sixth bar chart (red) shows the number of predicted effectors for each genome. The seventh bar plot (green) represents the number of predicted RxLR effectors among the predicted effectors, predicted by homology searching. The eight (orange) and the last one (blue) show the number of effectors containing the CRN motif and the number of effectors containing the WYL domain respectively.

Supplementary Figure 6 Box plots of FIRs of all genes, BUSCO genes and RxLRs in all the studied species. Here the positive FIR values were plotted with whisker = 0.2 parameter.
