# Supplementary figures and images for: Comparative Genome Analysis Across 128 Phytophthora Isolates Reveal Species-Specific Microsatellite Distribution and Localized Evolution of Compartmentalized Genomes
Source: Front Microbiol. 2022 Mar 16;13:806398. doi: 10.3389/fmicb.2022.806398 (PMC8967354; doi:10.3389/fmicb.2022.806398)

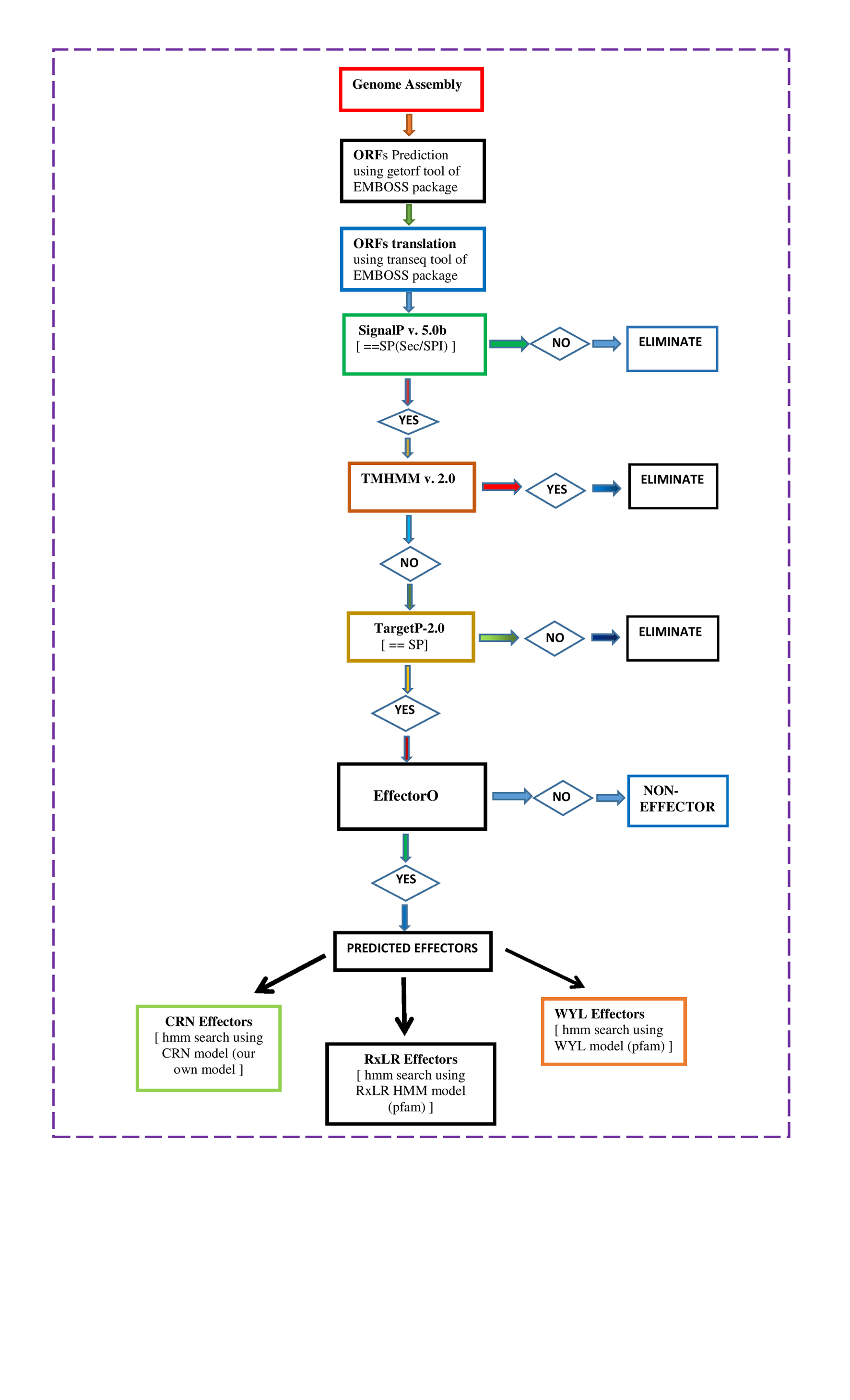

Supplement: Supplementary Figure 1 — Overall pipeline used for secretome and effector prediction. [file Image_1.PNG]

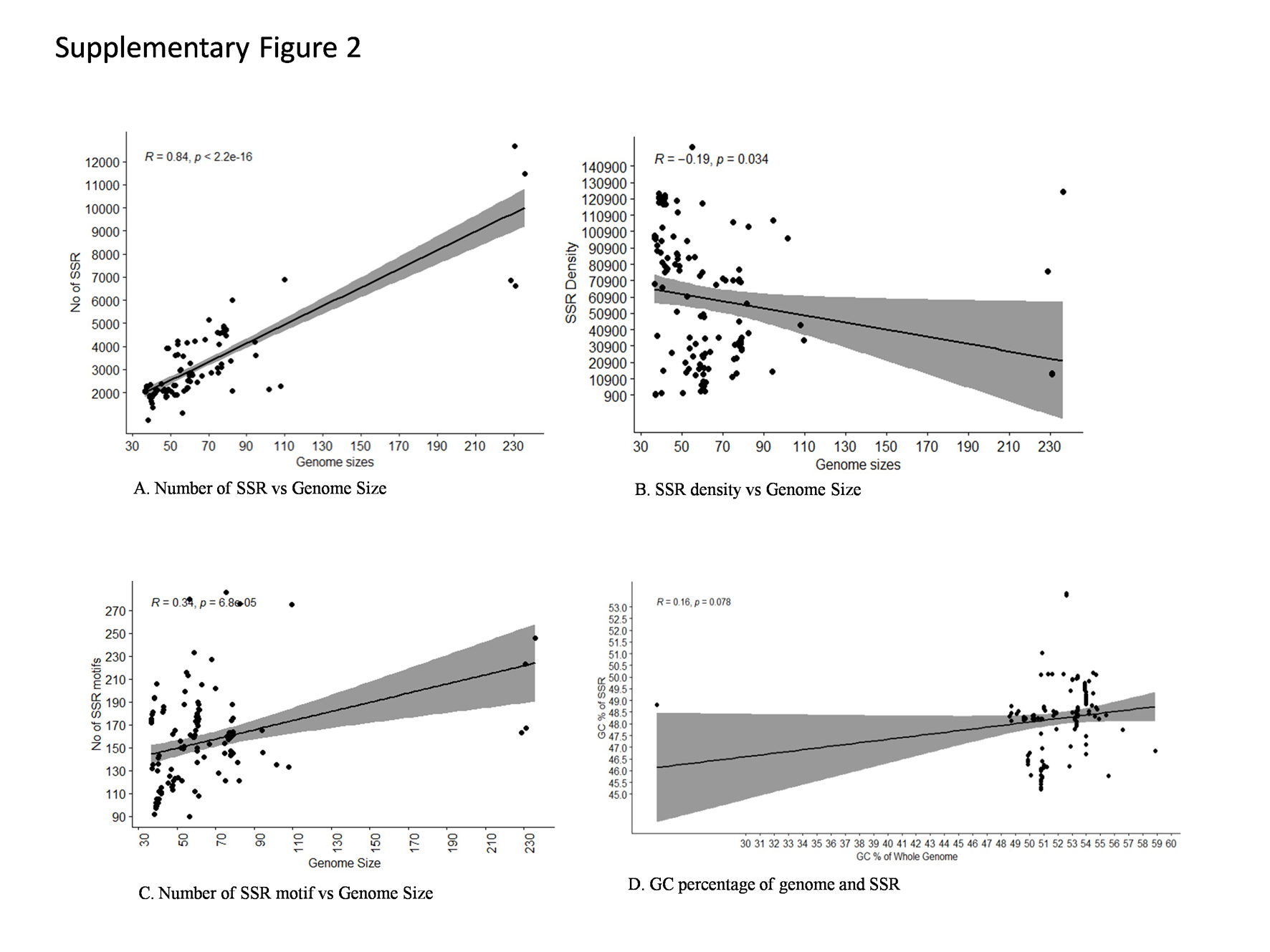

Supplement: Supplementary Figure 2 — (A) Pearson’s correlation coefficient of SSR number and Genome size. Result indicates number of SSRs positively correlated with genome size. (B) There was weak negative correlation between SSR density and genome size. (C) Number of SSR motifs shows weak negative correlation with genome size. (D) GC percentage of genome and SSR are positively correlated. [file Image_2.JPEG]

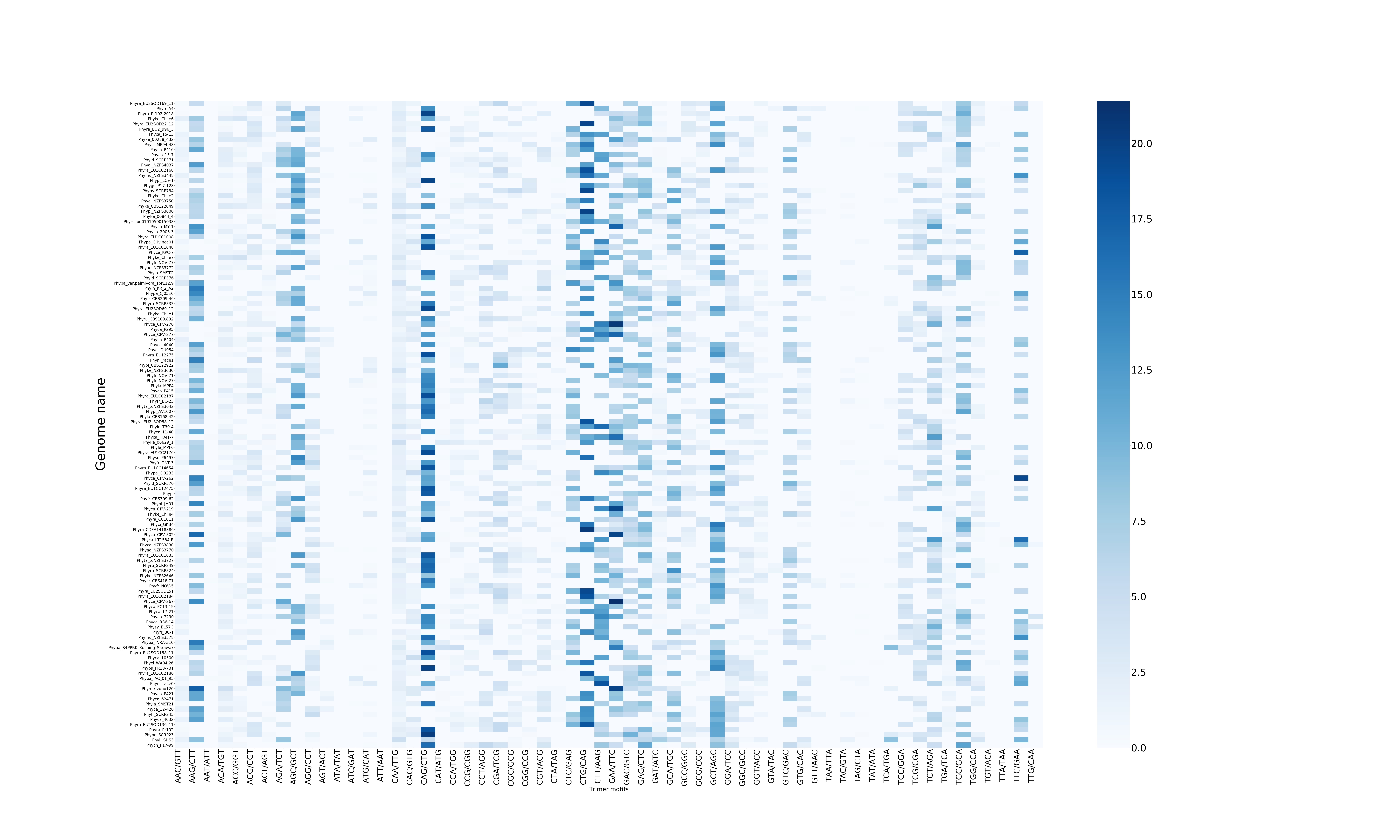

Supplement: Supplementary Figure 3 — Heatmap depicts the percentage frequency of trinucleotide SSR group motifs in the coding sequences (CDS). There is a clear abundance of CAG/CTG, AAG/CTT motifs. [file Image_3.JPEG]

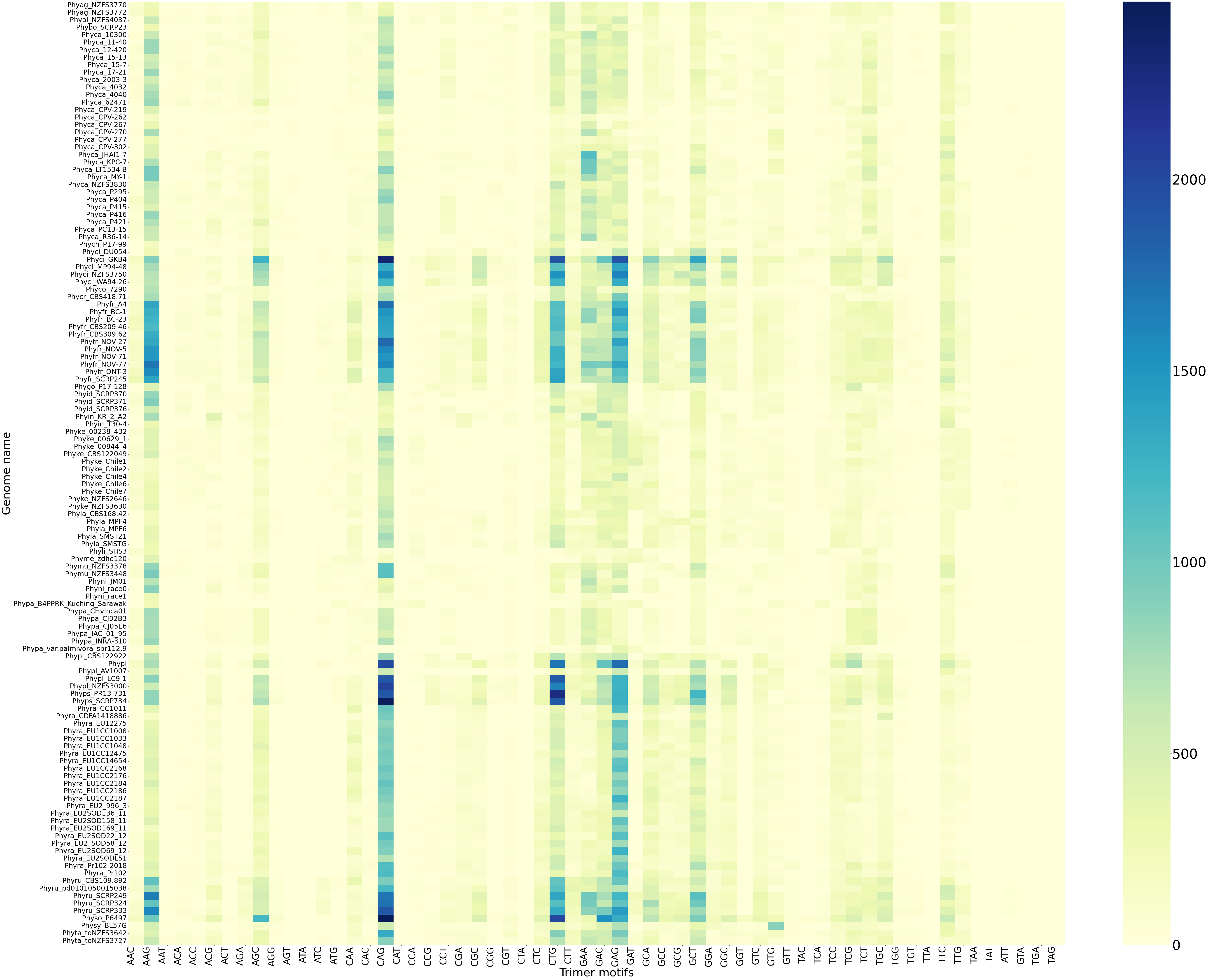

Supplement: Supplementary Figure 4 — Heatmap depicts the in-frame cumulative frequency of trinucleotide SSR motifs in the coding sequences (CDS). There is a clear dominance of CAG, CTG, AAG motifs. [file Image_4.PNG]

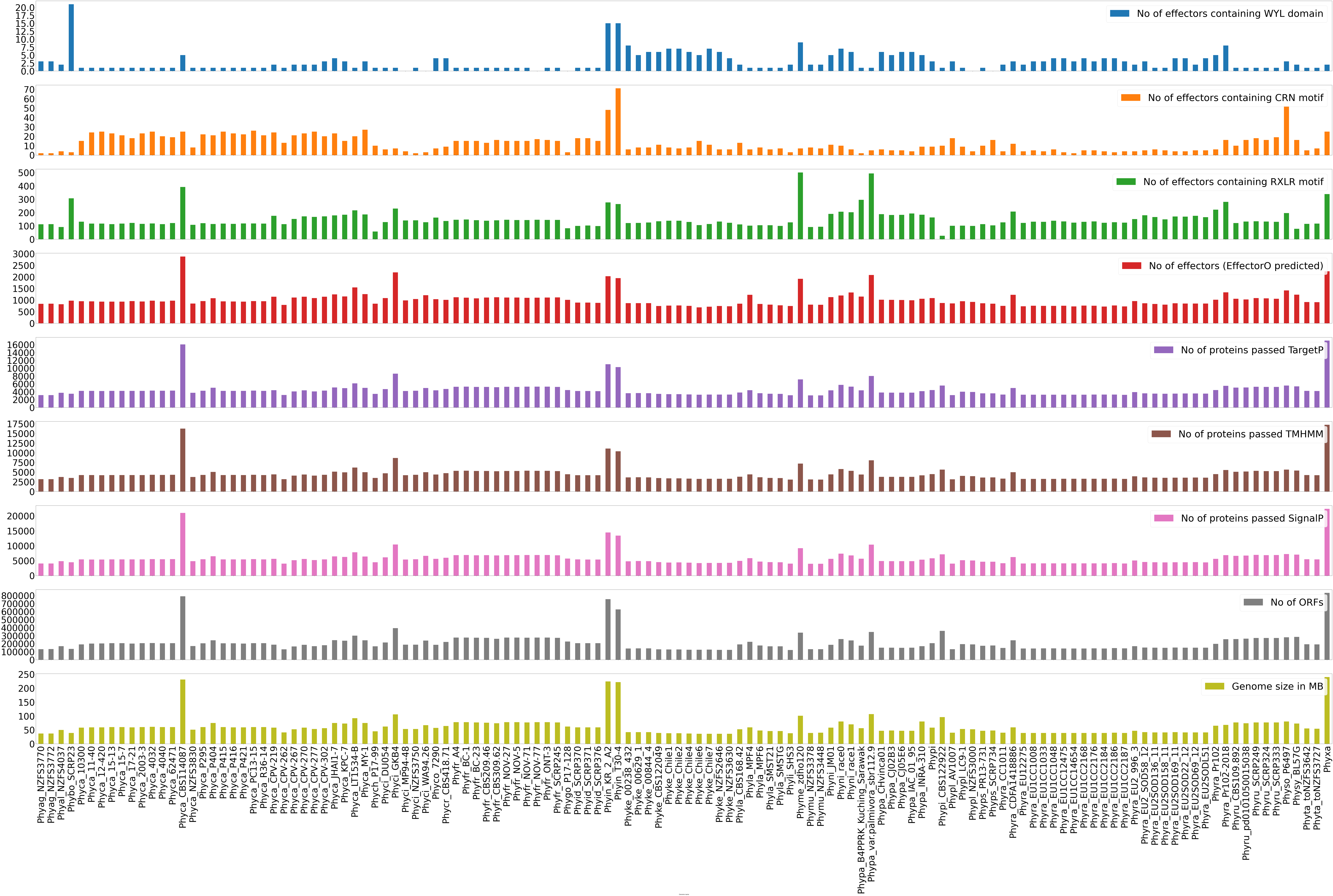

Supplement: Supplementary Figure 5 — Effector prediction pipeline containing the number of proteins filtered out in each step. The first bar chart (bottom most) (light green) represents the genome sizes of 128 Phytophthora isolates studied. The second bar chart (ash) represents the total number of Open reading frames (ORFs) predicted by getorf function of EMBOSS package. These ORFs are further translated in one frame and the translated sequences were used for secretome prediction. The third bar chart (pink) shows the number of secretory proteins containing signal peptide (SP), based on SignalP v. 5.0b prediction. The fourth one (brown) shows the number of secretory proteins retained after the TMHMM analysis that does not contain any TransMembrane Helices (TMHs). The fifth one (violet) shows the number of secretory proteins that passed TargetP analysis. These retained proteins after the TargetP are used for effector prediction using EffectorO. The sixth bar chart (red) shows the number of predicted effectors for each genome. The seventh bar plot (green) represents the number of predicted RxLR effectors among the predicted effectors, predicted by homology searching. The eight (orange) and the last one (blue) show the number of effectors containing the CRN motif and the number of effectors containing the WYL domain respectively. [file Image_5.PNG]

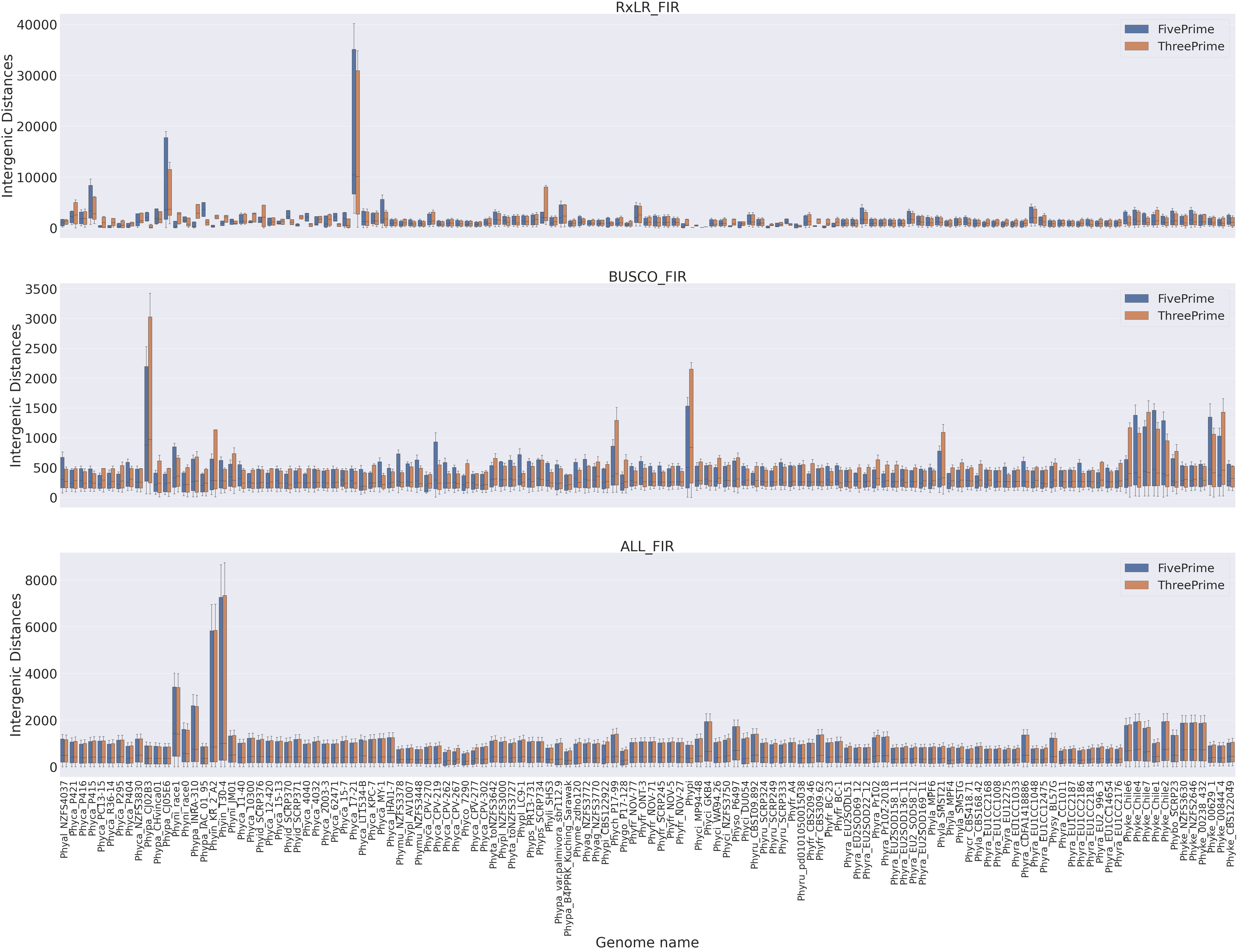

Supplement: Supplementary Figure 6 — Box plots of FIRs of all genes, BUSCO genes and RxLRs in all the studied species. Here the positive FIR values were plotted with whisker = 0.2 parameter. [file Image_6.PNG]
